# Diabetes IN develOpment (DINO): the bio-psychosocial, family functioning and parental well-being of youth with type 1 diabetes: a longitudinal cohort study design

**DOI:** 10.1186/s12887-015-0400-1

**Published:** 2015-07-15

**Authors:** Minke M. A. Eilander, Maartje de Wit, Joost Rotteveel, Henk Jan Aanstoot, Willie M. Bakker-van Waarde, Euphemia C. A. M. Houdijk, Marjolein Luman, Roos Nuboer, Jaap Oosterlaan, Per Winterdijk, Frank J. Snoek

**Affiliations:** Department of Medical Psychology, VU University Medical Center, De Boelenlaan 1117, 1081 HV Amsterdam, The Netherlands; EMGO+Institute for Health and Care Research, VU University Medical Center, Van der Boechorststraat 7, 1081 BT Amsterdam, The Netherlands; Department of Pediatrics, VU Medical Center, De Boelelaan 1118, 1081 HV Amsterdam, The Netherlands; Diabeter, Center for Pediatric and Adolescent Diabetes Care and Research, Blaak 6, 3011 TA Rotterdam, The Netherlands; Department Pediatrics, University Hospital of Groningen, Hanzeplein 1, 9700 RB Groningen, The Netherlands; Department of Pediatrics, Haga Hospital Juliana Children’s Hospital, Sportlaan 600, 2566 MJ the Hague, The Netherlands; Department Clinical Neuropsychology, Vrije Universiteit, Van der Boechorststraat 1, 1081 BT Amsterdam, The Netherlands; Department of Pediatrics, Meander Medical Centre, Maatweg 3, 3813 TZ Amersfoort, The Netherlands; Department of Medical Psychology, Academic Medical Center (AMC), Meibergdreef 9, 1100 DD Amsterdam, The Netherlands

**Keywords:** Type 1 diabetes, Development, Youth, Quality of life, Well-being, Adolescence, Cognition, HbA1c, Psychosocial, Parents

## Abstract

**Background:**

Strict glycemic control during adolescence decreases the risk of developing complications later in life, even if this level of control is not maintained afterwards. However, the majority of adolescents with type 1 diabetes (T1D) are in poor control and so far medical or psychological interventions have shown limited success. Adolescence is characterized by major biological, psychosocial, cognitive and parent–child relationship changes and the complex interaction between these developmental trajectories, and its impact on health outcomes is still poorly understood. A specific topic of interest in this context is the timing of diagnosis. The longitudinal study DINO (Diabetes IN develOpment) aims to examine:

1. If and how the onset of T1D before vs. during puberty results in different outcomes of glycemic control, self-management, psychological functioning and diabetes-related quality of life.
2. The timing of onset of disturbed eating behavior, its risk factors and its prospective course in relation to glycemic and psychological consequences.
3. If and how the onset of T1D before vs. during puberty results in different family functioning and parental well-being.
4. If and how the cognitive development of youth with T1D relates to glycemic control and diabetes self-management.

**Methods/design:**

DINO, a longitudinal multi-center cohort study is conducted in youth with T1D in the age range 8–15 years at baseline. Participants will be divided into two subgroups: pre-pubertal and pubertal. Both groups will be followed for 3 years with assessments based on a bio-psychosocial model of diabetes, scheduled at baseline, 12 months, 24 months and 36 months examining the biological, psychosocial -including disturbed eating behaviors- and cognitive development, family functioning and parental well-being.

**Discussion:**

A better understanding of how the different trajectories affect one another will help to gain insight in the protective and risk factors for glycemic outcomes and in who needs which support at what moment in time. First results are expected in 2016.

**Electronic supplementary material:**

The online version of this article (doi:10.1186/s12887-015-0400-1) contains supplementary material, which is available to authorized users.

## Background

**Fig. 1:**
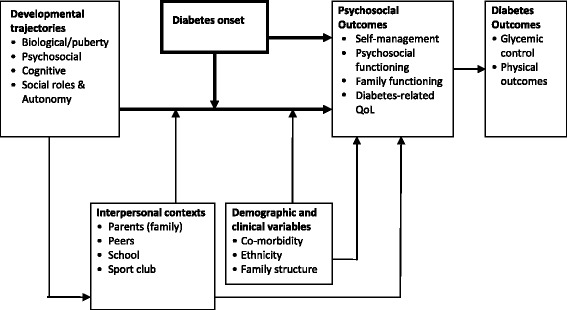
A framework for understanding the development and outcomes from pre-puberty into puberty. Bio-psychosocial model based on Holmbeck and Sherpa [74]

In 2009, a report in the Lancet concluded that: “If present trends continue, doubling of new cases of type 1 diabetes in European children younger than 5 years is predicted between 2005 and 2020, and prevalent cases younger than 15 years will rise by 70 %. Adequate health-care resources to meet these children’s needs should be made available” [1]. This clearly underscores the importance of understanding the specific (changing) needs of youth with type 1 diabetes (T1D) to improve quality and efficacy of pediatric diabetes care. This holds in particular for adolescent diabetes care, as clinical data have shown repeatedly that during adolescent years patients have great difficulty reaching and maintaining optimal glycemic control [2, 3]. Less than 15 % of the young patients keep constant or reach HbA_1c_ levels below 8 % (64 mmol/mol) from pre-puberty to young adulthood [2, 4]. In contrast to earlier belief, puberty years provide no protection against the risk of developing microvascular complications in later years as a result of prolonged hyperglycemia. In fact the reverse is true: Diabetes Control and Complications Trial/Epidemiology of Diabetes Interventions and Complications (DCCT/EDIC) Study has convincingly shown that the better the glycemic control during adolescence, the lower the risk of developing complications later on in life - even if that level of control is not maintained afterwards [5]. Adolescence is a critical period for the establishment of lifelong positive and risky health-related behaviors and, importantly, such ‘programming’ apparently applies to mental health as well [6]. In what manner biological, psychosocial and cognitive programming interact in youth with T1D is largely unknown. Of interest is the question how different trajectories develop during pre- and pubertal years, and to what extent these years offer a window of opportunity for early detection and targeted interventions to improve health outcomes. With the longitudinal cohort research DINO (Diabetes IN develOpment) the complex interaction of biological, psychosocial and cognitive development, family functioning and parental well-being will be studied. In order to do so, a bio-psychosocial approach is called for. The bio-psychosocial model appreciates these complex interactions, the onset and the demands of diabetes (Fig. 1).

### Biological development

There is substantial variation between individuals in the time of onset, duration and termination of the pubertal development and these differences have social and psychological consequences [7]. In addition is known that the onset and termination of puberty is delayed in children with diabetes compared to healthy youth [8, 9]. However, the consequences for diabetes self-management and health outcomes have not been studied. Research revealed that glycemic control tends to be better for children with shorter diabetes duration [10–12]. One study specifically showed that in T1D patients with pubertal compared to pre-pubertal onset, glycemic control was better and daily insulin doses were lower after 6 years of diabetes, irrespective of age-related factors [13]. Perhaps as a result of that, pre-pubertal onset of diabetes is found to predict earlier onset of retinopathy [14], suggesting that youth diagnosed during or after puberty do better than those diagnosed early in life. Older longitudinal studies showed that patients diagnosed before the age of 13 had better adherence to diabetes management over a 4 year follow-up period compared to patients diagnosed after this age [15]. Deterioration in adherence occurred in all age ranges as duration increased [10]. It is of note, that these longitudinal studies used age as an indicator for pubertal status and not the actual physical development such as Tanner stage, in which the based on primary and secondary sex characteristics is scaled [16]. Gender differences and individual variations in puberty onset were therefore not taken into account in these previous studies. Reviews have clearly identified the lack of prospective cohort studies in representative (pre)pubertal groups [17, 18]. Whether developing diabetes during puberty alters the duration and termination of puberty and results in psychological risks is unknown.

### Psychosocial development

In general, mid-adolescence appears the most vulnerable period for developing psychological problems [19]. In youth with diabetes, rates of depression, anxiety and disturbed eating behavior tend to be worrisomely elevated and are associated with poor glycemic control [20–23]. Adolescents with T1D have more emotional issues compared to healthy peers [24]. A study in female adolescents with diabetes showed that adolescents diagnosed <3 years from menarche [25], a lower overall sense of control was associated with poorer metabolic control. Hormonal fluctuations due to puberty can result in frequent ‘unexplainable’ (high) blood glucose values, easily inducing feelings of anger, frustration and discouragement, thereby contributing to poor adherence and subsequent deterioration of glycemic control and quality of life (QoL) [26]. Ten years after diagnosis, young adults with diabetes seem in general to be psychologically well adjusted, but do report lower perceived competence, including self-worth [27]. Low (diabetes specific) self-esteem is found to be associated with poor adherence and a predictor of deteriorating glycemic control in late adolescence [4, 28]. However, as stated before, the majority of these studies have not made a distinction between pre- and pubertal onset of diabetes and can therefore not inform us on the relevance of timing of diabetes onset on psychosocial development.

Adolescents with T1D are at an increased risk of disturbed eating behavior (DEB) compared to healthy peers [29–33] due to hormonal changes [3], the focus on food, issues around control and autonomy in diabetes care. This ‘Diabulimia’ has been frequently reported among adolescents with T1D; 33–53 % reported to engage in unhealthy eating behaviors and insulin restriction for weight purposes was prevalent in up to 30 % of patients [31, 34–39]. DEB increases the risk for poorer glycemic control, earlier complications from diabetes, particularly retinopathy and nephropathy as well as mortality [32–37, 40–43]. In T1D, it is suggested that in most cases the DEB developed after diabetes onset [44]. Although the peak of onset of DEB is in adolescence only one study assessed risk factors for the onset of DEB in adolescent girls [45]. Currently, diabetes teams are hesitant to discuss DEB with their patients [46], because they are afraid they might bring the association between insulin and weight control to mind of the adolescent. It is important to know the timing of onset of DEB, who is at risk, how to address these behaviors and to be able to identify those at risk for DEB [40].

### Cognitive development

Neuropsychological research shows that children with T1D, especially those with early-onset diabetes (≤6 years of age), have mild impairment of cognitive functioning [47] including poorer academic achievement [48], lower verbal intelligence, and worse performance on measures of attention, executive function, mental flexibility, and psychomotor speed [47] compared to healthy controls. This challenges adolescents’ diabetes adherence behaviors, since these tasks are of great importance to organize and plan the diabetes management.

In general, adolescence is a critical period for brain maturation, essential for the development of higher cognitive functions. Significant improvements in cognitive processing speed and intellectual functioning are evident throughout adolescence and mature in young adulthood, with the most dramatic improvements occurring in the development of executive functions including abstract thought, organization, decision making, planning, and response inhibition [19, 49]. This implies that later on in adolescence the ability to critically outweigh the costs and benefits of (non) adherence behaviors increases [50]. However, until that time the adolescent’s brain is inclined to engage in risk taking behavior and prefers immediate rather than long term satisfaction [49]. Given their stage of cognitive development and the challenges facing adolescents might challenge their ability to manage their diabetes on a daily basis.

### Family functioning and parental well-being

The way the family of T1D youth functions is important, both as determinant and consequent of poor diabetes control. A negotiating parent–child environment is beneficial for children with diabetes. In addition, shared responsibility for diabetes management tasks is shown to be associated with better psychological health, self-care behavior and metabolic control [51–53]. A lack of collaboration between children and parents can result in conflicts which are often associated with poor glycemic control and QoL [54, 55], however, this seems to be related to ethnicity [56]. Shared responsibility regarding diabetes tasks between parents and adolescents (rather than complete/sudden transfer of parental control) for diabetes management may serve as a way to achieve autonomy for self-care. Youth with an inordinate self-care autonomy relative to their psychological maturity are at greater risk of poor treatment adherence, worse diabetic control and more hospitalizations [57]. Inconsistencies regarding competence and independence between parents and children with T1D is associated with poorer diabetes outcomes [58]. Furthermore, the better parents are able to adopt youth’s perspectives the better the glycemic control [59]. Recent research reveals that parental involvement [60] in diabetes care and greater overall parental support [61] are associated with better health [60] and service use [61], and greater parental motivation is related to child’s healthier diet [62]. These findings highlight the importance of parenting practices. One of the major tasks for parents is to be responsive to adolescents’ needs for increasing responsibility and decision making power while at the same time maintaining a high level of cohesiveness in the family. However, parental well-being influences the way this task proceeds. Recent research reveals that parents with T1D children were more anxious and perceived less family cohesion than the parents of healthy youth [63]. The diagnosis, hypoglycemic events, as well as the chronic nature of diabetes and its demands all contribute to anxiety and depressive symptoms in parents [64, 65]. Importantly, worse parental well-being is shown to be associated with poorer glycemic control of the children [66, 67] and maternal depression is found to be associated with acute hospitalization [68]. Of interest is how family functioning and parental well-being influences adolescents’ diabetes outcomes and development, and how parental well-being influences youths’ diabetes and psychosocial outcomes.

Overall, studies integrating the biological, psychosocial and cognitive developmental trajectories, family functioning and parental well-being are lacking [18] with a few positive exceptions. Wiebe *et al.* examined the relationship between self-efficacy, parental responsibilities, pubertal maturation and adherence [69]. Luyckx *et al.* determined developmental classes of glycemic control in young people with T1D throughout adolescence and emerging adulthood, in relation to general family climate and self-concept [4]. King *et al.* used latent growth class analysis to look at trajectories of metabolic control in relation to autonomy, diabetes management and hospitalizations [60]. These studies used a person-centered approach that is uniquely suited to capture diversification in glycemic control, looking for meaningful subgroups characterized by unique developmental pathways.

### Overall aims and research question

There is paucity of evidence with regard to the question if and how (living with) diabetes during pre-pubertal years and early adolescence predict glycemic control, self-management, psychosocial functioning and diabetes-related QoL during later years. The importance of being diagnosed with diabetes before versus during puberty has hardly received attention in past decades, while the mechanisms and role of puberty could give important information for (well-timed) future interventions.

The primary goal of DINO is to further our understanding how the onset of diabetes impacts the biological, psychosocial and cognitive development and family functioning and parental well-being during pre-pubertal and pubertal years in Dutch youth with T1D. With DINO we will examine:

1. If and how the onset of T1D before vs. during puberty results in different outcomes of glycemic control, self-management, psychological functioning and diabetes-related quality of life.
2. The timing of onset of disturbed eating behavior, its risk factors and its prospective course in relation to glycemic and psychological consequences.
3. If and how the onset of T1D before vs. during puberty results in different family functioning and parental well-being.
4. If and how the cognitive development of youth with T1D relates to glycemic control and diabetes self-management.

## Methods/Design

**Fig. 2:**
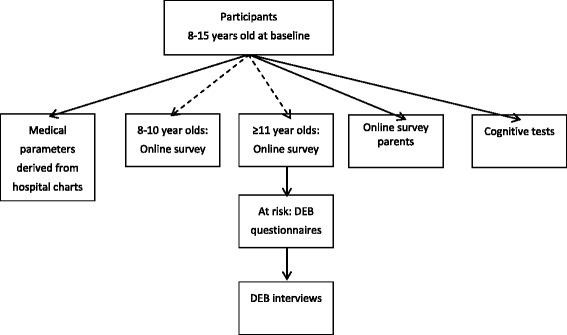
Flowchart annual DINO procedure

A prospective multi-center cohort study will be conducted in youth with T1D in the age range 8–15 years at baseline. For 3 years, participants’ biological, psychosocial and cognitive development will annually be assessed (at baseline, 12 months, 24 months and 36 months), as represented in Fig. 2. During the course of the study, newly diagnosed youth will be included 6 months after diagnosis -when diabetes regimen is habituated- and follow the scheduled assessments. By consequence, not all newly diagnosed will have the same number of assessments. Participants will be divided into two subgroups:

A) Pre-Pubertal Onset of Diabetes (Tanner stage 1) and
B) Pubertal Onset of Diabetes (Tanner stage 2–5).

Group A will provide information on the effects of puberty on the developmental trajectories in relation to diabetes outcomes in youth already diagnosed with diabetes. Group B will provide information on how the onset of diabetes affects the developmental trajectories.

### Procedure and participants

Five Dutch pediatric diabetes care clinics agreed to participate. Pediatricians will recruit youth diagnosed with T1D and their parents. Exclusion criteria are: other types of diabetes than Type 1 (e.g. type 2 or MODY), younger than 8 years or older than 15 years at baseline, not speaking the Dutch language, and mental retardation. All medical parameters are taken from hospital charts and no extra tests will be performed. As represented in Fig. 2, youth between 8 and 15 years at baseline with T1D who consent to participate will complete an online survey regarding their psychosocial development. If a paper survey is preferred or participants do not respond to the e-mail, a paper version is sent to their home address. Due to the age difference, 8–11 year olds will complete a shorter and more simple survey than participants 11 years and older. To gain better insight in the perspectives of the adolescents about DEB, we will conduct interviews with a selection of youth. Only youth at risk (based on their answers on the online survey) will be invited for the interview. The interviews will take place at the adolescent’s residence or at the outpatient clinic, depending on the adolescent’s preference. Parents will report on family functioning and parental well-being by an online survey as well, unless the paper version is preferred. A neuropsychological test battery will be used to assess the cognitive development. Most test results will be compared to normative data, however, three measures will also be administered to a gender and age matched sample of 100 healthy controls as reference values are not yet available for these tasks. These healthy controls are derived from schools in the Netherlands and will be measured cross-sectional.

### Ethical considerations

The study protocol was approved by the medical ethical committee of VU University Medical Centre (date: December 19th, 2012). Youth and parents are provided with written information about the study and are asked to provide written informed consent (both parents -if applicable- and youth ≥12) prior to the data collection.

### Study measures

An overview of study measures is shown in Tables 1, 2 and 3.

**Table 1:** Overview of study measures – Socio-Demographic and clinical data

	History	Baseline	12 months	24 months	36 months
Socio-Demographic data					
Date of birth		H			
Gender		H			
Ethnicity		P			
Education level child		P + C	P + C	P + C	P + C
Socioeconomic status		P			
Family structure		C	C	C	C
Family related life events	P	P	P	P	P
Clinical data					
History of medical and psychological co-morbidity	H	H	H	H	H
Treatment regime		H	H	H	H
Care consumption	H	H	H	H	H
Tanner stage at time of diagnosis [16]		H			
Current Tanner stage [16]		H	H	H	H
Blood pressure		H	H	H	H
Weight and Height		H	H	H	H
Hemoglobin A1c (HbA1c)	H	H	H	H	H
Number of diabetes related hospitalizations	H	H	H	H	H
DKA	H	H	H	H	H
Indicators for complications	H	H	H	H	H
Severe hypoglycemic episodes	P	P	P	P	P

*H* Hospital, *P* Parent, *C* Child, *HC* Healthy control

**Table 2:** Overview of study measures – Psychosocial development, DEB, Cognitive development

Psychosocial development					
Strengths and Difficulties Questionnaire (SDQ) [75]		P + C ≥11	P + C ≥11	P + C ≥11	P + C ≥11
Revised Children’s Quality of Life Questionnaire (KINDL-R) self esteem subscale [76]		C	C	C	C
KIDSCREEN Autonomy subscale [77, 78]		C ≥11	C ≥11	C ≥11	C ≥11
Diabetes Family Responsibility Questionnaire (DFRQ) [79]		P + C ≥11	P + C ≥11	P + C ≥11	P + C ≥11
MIND Youth Questionnaire (MY-Q) [80]		C	C	C	C
Adapted version for 8–10 year olds					
Confidence in Diabetes Self-care Youth (CIDS-youth) [81]		C ≥11	C ≥11	C ≥11	C ≥11
Mismanagement scale – renewed [82]		C ≥11	C ≥11	C ≥11	C ≥11
Adherence	H	H	H	H	H
Disturbed Eating Behavior (DEB)^a^					
2 questions regarding dieting status and frequency			C ≥11	C ≥11	C ≥11
Diabetes Eating Problems Scale-Revised (DESP-R) [36, 43]			C ≥11	C ≥11	C ≥11
Questions of the AHEAD study [35]			C ≥11	C ≥11	C ≥11
DEB semi structured interview			C ≥11		
2 MY-Q subscale body and weight [80]			P	P	P
Cognitive development					
Wechsler Intelligence Scale for Children III (WISC-III) subtests Information; Picture Arrangement; Arithmetic; Block Design; Digit Span [83, 84]		C + HC	C	C	C
Attention Network Task (ANT)-adapted version [85, 86]		C + HC	C	C	C
Eriksen Flanker Task [87, 88]		C + HC	C	C	C
Klingberg Task – adapted version [86, 89, 90]		C + HC	C	C	C
Behavior Rating Inventory of Executive Functioning questionnaire (BRIEF) [91, 92]		C + HC	P	P	P

*H* Hospital, *P* Parent, *C* Child, *HC* Healthy control

^a^DEB is assessed in a step-wise manner in order to minimize the burden in adolescents with no DEB and younger participants. Kindly note the online text Additional file 1

**Table 3:** Overview of study measures – parental assessment

Parental assessment				
Problem Areas In Diabetes-Parents Revised (PAID-PR) [93, 94]	P	P	P	P
WHO-Five Well-being Index (WHO-5) [95–97]	P	P	P	P
Diabetes Family Behavior Checklist (DFBC) [98]	P	P	P	P

*H* Hospital, *P* Parent, *C* Child, *HC* Healthy control

A full description of these measures is presented in the online text Additional file 1.

### Data analyses

Using descriptive statistics, baseline data are analyzed cross-sectionally and scores are compared with reference values when applicable. Uni-variate analysis ANOVA will be used to explore differences between boys and girls and Tanner stages. Pearson or Spearman correlation is used to explore associations between several outcomes (such as age, cognitive development, social-emotional development, glycemic control, psychological functioning, QoL, diabetes management and DEB). To examine whether associations are mediated by other variables, multiple mediation analysis will be used [70].

With regard to the cognitive development, performance on the computerized measures will be administered to a gender and age matched sample of 100 healthy controls. Because of the longitudinal nature of this study, Generalized Estimation Equations (GEE) is used to examine the differences between the two groups diagnosis pre-puberty vs puberty on glycemic control, self-management, psychological functioning and diabetes related QoL. The differences between the two groups on family functioning and parental well-being and the development of DEB are investigated by GEE as well. GEE adjusts for the correlation between repeated observations taken in the same patient and has the advantage of handling longitudinal data on subjects with varying numbers of unequally spaced observations. The latter is important, because the assessments are scheduled within routine care and as a consequence, the time between the consultations will differ. Longitudinal linear regression analyses, using GEE, enables us to examine the association between the developmental trajectories in relation to diabetes onset and outcomes.

Data are controlled for demographic and clinical variables and examined for associations with and predictors of biological and social-emotional developmental outcomes. Latent class growth analysis is used to identify developmental trajectories of glycemic control and psychological, cognitive and family functioning.

For the analyses of the interviews, a framework approach is used [71, 72]: interviews are transcribed verbatim and key words and codes extract content from the text is assigned by two researchers, at least for the first few transcripts using Atlas.Ti.

### Sample size

Sample size calculations indicate that a sample of 86 patients is sufficient to detect a statistically clinically difference of ≥ 0.5 % HbA_1c_ (sd = 1.1 %) at a significance level of 5 % with a power of 80 %, given three follow-up measurements using GEE analyses and taking into account the within-subject correlation (rho = 0.7). Sample size calculations for psychological functioning (as measured with the SDQ) indicate that a sample of 40 patients is sufficient to detect a difference of 20 % in the proportion likely “cases” with mental health disorders (assuming *p* = 0.33 [73]) at a significance level of 5 % with a power of 80 %, given three follow-up measurements using longitudinal logistic regression analyses and taking into account the within-subject correlation (rho = 0.2). This means that in order to detect differences in HbA_1c_ and likely cases of psychological dysfunction between youth with pre vs. pubertal onset of diabetes, both groups should contain at least 43 patients. Given an expected drop-out rate of 10 %, we will include at least an additional 4 patients in each group. Therefore, we aim to include a minimum of 100 boys and 100 girls (*N* = 200).

## Discussion

To the best of our knowledge, only a few studies have examined the effect of diabetes onset during pubertal vs. pre-pubertal years and little longitudinal research is available in children and teenagers with T1D, although a lot changes during pubertal years. There is large individual variability in glycemic and psychological trajectories. The way youth and families cope with puberty and the developmental changes differ as well. In our research project DINO we aim to assess the different developmental trajectories (biological, psychosocial -including disturbed eating behaviors- and cognitive) and family functioning and parental well-being. This will provide insight in protective and risk factors for glycemic outcomes and in who needs which support at what moment in time. Better understanding contributes to the optimization of pediatric diabetes care. This might include the use of more sensitive screening instruments, for example to assess cognitive functioning in relation to self-management, risk factors for DEB and other psychological problems. This would enable diabetes teams to better personalize their care for adolescents with diabetes. Better understanding can contribute to the development of new interventions aimed at, for example, prevention and/or treatment of depressive symptoms and better tailoring of self-management education to the developmental phase of the child. First results of DINO are expected in 2016.

### Limitations of this comprehensive study

A selection bias and the adolescents lost to follow up might influence the external validity of the results. However, this is almost unavoidable in longitudinal research studies. In addition, the newly diagnosed youth are not followed the entire 3 years of the study. To assess psychological development and family functioning and parental well-being self-report measures are used. With regard to the cognitive development, there can be an interviewers or observers bias as multiple test leaders will perform the neuropsychological assessments throughout the Netherlands. With a standardized training program we try to minimize the bias. The use of neuroimaging techniques such as functional Magnetic Resonance Imaging (fMRI) might provide a more objective, additional source of information, nonetheless this was not an option within the current budget. As seen in the bio-psychosocial model (Fig. 1) the T1D adolescent functions in a broad social network. School and friends for example play an important role in youth’s development, yet we did not include these factors because of the feasibility of the study.

## Additional file

Additional file 1:
**Study measures online text supplement.**

## Abbreviations

T1D: Type 1 diabetes
DCCT/EDIC: Diabetes Control and Complications Trial/Epidemiology of Diabetes Interventions and Complications
DINO: Diabetes IN develOpment
QoL: Quality of Life
DEB: Disturbed eating behavior
HbA1c: Hemoglobin A1c
SDQ: Strengths and difficulties questionnaire
KINDL-R: Revised children’s quality of life questionnaire
DFRQ: Diabetes family responsibility questionnaire
MY-Q: MIND youth questionnaire
CIDS-youth: Confidence in diabetes self-care youth
DESP-R: Diabetes eating problems scale-revised
WISC-III: Wechsler Intelligence Scale for Children-III
ANT: Attention network task
BRIEF: Behavior rating inventory of executive functioning questionnaire
PAID-PR: Problem areas in diabetes-parents revised
WHO-5: WHO-five well-being index
DFBC: Diabetes family behavior checklist

## References

[1] Patterson CC, Dahlquist GG, Gyurus E, Green A, Soltesz G (2009). Incidence trends for childhood type 1 diabetes in Europe during 1989–2003 and predicted new cases 2005–20: a multicentre prospective registration study. Lancet.

[2] Dabadghao P, Vidmar S, Cameron FJ (2001). Deteriorating diabetic control through adolescence-do the origins lie in childhood?. Diabet Med.

[3] Amiel SA, Sherwin RS, Simonson DC, Lauritano AA, Tamborlane WV (1986). Impaired insulin action in puberty. A contributing factor to poor glycemic control in adolescents with diabetes. N Engl J Med.

[4] Luyckx K, Seiffge-Krenke I (2009). Continuity and change in glycemic control trajectories from adolescence to emerging adulthood: relationships with family climate and self-concept in type 1 diabetes. Diabetes Care.

[5] White NH, Cleary PA, Dahms W, Goldstein D, Malone J, Tamborlane WV (2001). Beneficial effects of intensive therapy of diabetes during adolescence: outcomes after the conclusion of the Diabetes Control and Complications Trial (DCCT). J Pediatr.

[6] Bryden KS, Peveler RC, Stein A, Neil A, Mayou RA, Dunger DB (2001). Clinical and psychological course of diabetes from adolescence to young adulthood: a longitudinal cohort study. Diabetes Care.

[7] Tanner JM (1971). Sequence, tempo, and individual variation in the growth and development of boys and girls aged twelve to sixteen. Daedalus.

[8] Elamin A, Hussein O, Tuvemo T (2006). Growth, puberty, and final height in children with type 1 diabetes. J Diabetes Complicat.

[9] Rohrer T, Stierkorb E, Heger S, Karges B, Raile K, Schwab KO (2007). Delayed pubertal onset and development in German children and adolescents with type 1 diabetes: cross-sectional analysis of recent data from the DPV diabetes documentation and quality management system. Eur J Endocrinol.

[10] Kovacs M, Goldston D, Obrosky DS, Iyengar S (1992). Prevalence and predictors of pervasive noncompliance with medical treatment among youths with insulin-dependent diabetes mellitus. J Am Acad Child Adolesc Psychiatry.

[11] Alan MJ, Hauser ST, Lavori P, Wolfsdorf JI, Herskowitz RD, Milley JE (1990). Adherence among children and adolescents with insulin-dependent diabetes mellitus over a four-year longitudinal foliow-up: I. The influence of patient coping and adjustment. J Pediatr Psychol.

[12] Rosenbauer J, Dost A, Karges B, Hungele A, Stahl A, Bachle C (2012). Improved metabolic control in children and adolescents with type 1 diabetes A trend analysis using prospective multicenter data from Germany and Austria. Diabetes Care.

[13] Kordonouri O, Danne T, Enders I, Weber B (1998). Does the long-term clinical course of type I diabetes mellitus differ in patients with prepubertal and pubertal onset? Results of the Berlin Retinopathy Study. Eur J Pediatr.

[14] Donaghue KC, Fairchild JM, Craig ME, Chan AK, Hing S, Cutler LR (2003). Do all prepubertal years of diabetes duration contribute equally to diabetes complications?. Diabetes Care.

[15] Jacobson AM, Hauser ST, Lavori P, Wolfsdorf JI, Herskowitz RD, Milley JE (1990). Adherence among children and adolescents with insulin-dependent diabetes mellitus over a four-year longitudinal follow-up: I. The influence of patient coping and adjustment. J Pediatr Psychol.

[16] Tanner JM (1986). Normal growth and techniques of growth assessment. Clin Endocrinol Metab.

[17] Chida Y, Hamer M (2008). An association of adverse psychosocial factors with diabetes mellitus: a meta-analytic review of longitudinal cohort studies. Diabetologia.

[18] Sawyer SM, Drew S, Yeo MS, Britto MT (2007). Adolescents with a chronic condition: challenges living, challenges treating. Lancet.

[19] Holmbeck GN, Abad M, Friedman D, Jandasek B, Wolfe DA, Mash EJ (2006). Development and Psychopathology in Adolescents. Behavioral and Emotional Disorders in Adolescents: Nature, Assessment, and Treatment.

[20] Dantzer C, Swendsen J, Maurice-Tison S, Salamon R (2003). Anxiety and depression in juvenile diabetes: a critical review. Clin Psychol Rev.

[21] Blanz BJ, Rensch-Riemann BS, Fritz-Sigmund DI, Schmidt MH (1993). IDDM is a risk factor for adolescent psychiatric disorders. Diabetes Care.

[22] Lawrence JM, Standiford DA, Loots B, Klingensmith GJ, Williams DE, Ruggiero A (2006). Prevalence and correlates of depressed mood among youth with diabetes: the SEARCH for Diabetes in Youth study. Pediatrics.

[23] Hassan K, Loar R, Anderson BJ, Heptulla RA (2006). The role of socioeconomic status, depression, quality of life, and glycemic control in type 1 diabetes mellitus. J Pediatr.

[24] Zenlea IS, Mednick L, Rein J, Quinn M, Wolfsdorf J, Rhodes ET (2014). Routine behavioral and mental health screening in young children with type 1 diabetes mellitus. Pediatr Diabetes.

[25] Schwartz SA, Weissberg-Benchell J, Perlmuter LC (2002). Personal control and disordered eating in female adolescents with type 1 diabetes. Diabetes Care.

[26] Anderson BJ (2004). Family conflict and diabetes management in youth: clinical lessons from child development and diabetes research. Diabetes Spectrum.

[27] Jacobson AM, Hauser ST, Willett JB, Wolfsdorf JI, Dvorak R, Herman L (1997). Psychological adjustment to IDDM: 10-year follow-up of an onset cohort of child and adolescent patients. Diabetes Care.

[28] Schneider S, Iannotti RJ, Nansel TR, Haynie DL, Sobel DO, Simons-Morton B (2009). Assessment of an illness-specific dimension of self-esteem in youths with type 1 diabetes. J Pediatr Psychol.

[29] Goebel-Fabbri A (2009). Disturbed eating behaviors and eating disorders in type 1 diabetes: clinical significance and treatment recommendations. Curr Diab Rep.

[30] Rodin G, Olmsted MP, Rydall AC, Maharaj SI, Colton PA, Jones JM (2002). Eating disorders in young women with type 1 diabetes mellitus. J Psychosom Res.

[31] Colton P, Olmsted M, Daneman D, Rydall A, Rodin G (2004). Disturbed eating behavior and eating disorders in preteen and early teenage girls with type 1 diabetes: a case-controlled study. Diabetes Care.

[32] Mannucci E, Rotella F, Ricca V, Moretti S, Placidi GF, Rotella CM (2005). Eating disorders in patients with type 1 diabetes: a meta-analysis. J Endocrinol Invest.

[33] Young V, Eiser C, Johnson B, Brierley S, Epton T, Elliott J (2013). Eating problems in adolescents with type 1 diabetes: a systematic review with meta-analysis. Diabet Med.

[34] Rydall AC, Rodin GM, Olmsted MP, Devenyi RG, Daneman D (1997). Disordered eating behavior and microvascular complications in young women with insulin-dependent diabetes mellitus. N Engl J Med.

[35] Neumark-Sztainer D, Patterson J, Mellin A, Ackard DM, Utter J, Story M (2002). Weight control practices and disordered eating behaviors among adolescent females and males with type 1 diabetes associations with sociodemographics, weight concerns, familial factors, and metabolic outcomes. Diabetes Care.

[36] Wisting L, Froisland DH, Skrivarhaug T, Dahl-Jorgensen K, Ro O (2013). Disturbed eating behavior and omission of insulin in adolescents receiving intensified insulin treatment A nationwide population-based study. Diabetes Care.

[37] Goebel-Fabbri AE, Fikkan J, Franko DL, Pearson K, Anderson BJ, Weinger K (2008). Insulin restriction and associated morbidity and mortality in women with type 1 diabetes. Diabetes Care.

[38] Bryden KS, Neil A, Mayou RA, Peveler RC, Fairburn CG, Dunger DB (1999). Eating habits, body weight, and insulin misuse. A longitudinal study of teenagers and young adults with type 1 diabetes. Diabetes Care.

[39] Peveler RC, Bryden KS, Neil HA, Fairburn CG, Mayou RA, Dunger DB (2005). The relationship of disordered eating habits and attitudes to clinical outcomes in young adult females with type 1 diabetes. Diabetes Care.

[40] Young-Hyman DL, Davis CL (2010). Disordered eating behavior in individuals with diabetes: importance of context, evaluation, and classification. Diabetes Care.

[41] Takii M, Uchigata Y, Tokunaga S, Amemiya N, Kinukawa N, Nozaki T (2008). The duration of severe insulin omission is the factor most closely associated with the microvascular complications of Type 1 diabetic females with clinical eating disorders. Int J Eat Disord.

[42] d’Emden H, Holden L, McDermott B, Harris M, Gibbons K, Gledhill A (2013). Disturbed eating behaviours and thoughts in Australian adolescents with type 1 diabetes. J Paediatr Child Health.

[43] Markowitz JT, Butler DA, Volkening LK, Antisdel JE, Anderson BJ, Laffel LM (2010). Brief screening tool for disordered eating in diabetes internal consistency and external validity in a contemporary sample of pediatric patients with type 1 diabetes. Diabetes Care.

[44] Cameron FJ, Northam EA, Ambler GR, Daneman D (2007). Routine psychological screening in youth with type 1 diabetes and their parents A notion whose time has come?. Diabetes Care.

[45] Olmsted MP, Colton PA, Daneman D, Rydall AC, Rodin GM (2008). Prediction of the onset of disturbed eating behavior in adolescent girls with type 1 diabetes. Diabetes Care.

[46] Tierney S, Deaton C, Whitehead J (2009). Caring for people with type 1 diabetes mellitus engaging in disturbed eating or weight control: a qualitative study of practitioners’ attitudes and practices. J Clin Nurs.

[47] Gaudieri PA, Chen R, Greer TF, Holmes CS (2008). Cognitive function in children with type 1 diabetes: a meta-analysis. Diabetes Care.

[48] Dahlquist G, Kallen B (2007). School marks for Swedish children whose mothers had diabetes during pregnancy: a population-based study. Diabetologia.

[49] Schiebener J, Garcia-Arias M, Garcia-Villamisar D, Cabanyes-Truffino J, Brand M (2014). Developmental changes in decision making under risk: the role of executive functions and reasoning abilities in 8-to 19-year-old decision makers. Child Neuropsychology.

[50] Holmbeck GN (2002). A developmental perspective on adolescent health and illness: an introduction to the special issues. J Pediatr Psychol.

[51] Helgeson VS, Reynolds KA, Siminerio L, Escobar O, Becker D (2008). Parent and adolescent distribution of responsibility for diabetes self-care: links to health outcomes. J Pediatr Psychol.

[52] Anderson BJ, Brackett J, Ho J, Laffel LM (1999). An office-based intervention to maintain parent-adolescent teamwork in diabetes management. Impact on parent involvement, family conflict, and subsequent glycemic control. Diabetes Care.

[53] Wiebe DJ, Berg CA, Korbel C, Palmer DL, Beveridge RM, Upchurch R (2005). Children’s appraisals of maternal involvement in coping with diabetes: enhancing our understanding of adherence, metabolic control, and quality of life across adolescence. J Pediatr Psychol.

[54] de Wit M, Delemarre-van de Waal H, Bokma JA, Haasnoot K, Houdijk MC, Gemke RJ (2008). Monitoring and discussing health-related quality of life in adolescents with type 1 diabetes improve psychosocial well-being: a randomized controlled trial. Diabetes Care.

[55] Anderson BJ, Brackett J, Snoek FJ, Skinner TC (2005). Diabetes in Children. Psychology in Diabetes Care.

[56] Main A, Wiebe DJ, Croom AR, Sardone K, Godbey E, Tucker C (2014). Associations of parent-adolescent relationship quality with type 1 diabetes management and depressive symptoms in Latino and Caucasian Youth. J Pediatr Psychol.

[57] Wysocki T, Taylor A, Hough BS, Linscheid TR, Yeates KO, Naglieri JA (1996). Deviation from developmentally appropriate self-care autonomy: association with diabetes outcomes. Diabetes Care.

[58] Butner J, Berg CA, Osborn P, Butler JM, Godri C, Fortenberry KT (2009). Parent-adolescent discrepancies in adolescents’ competence and the balance of adolescent autonomy and adolescent and parent well-being in the context of type 1 diabetes. Dev Psychol.

[59] Blicke M, Korner U, Nixon P, Salgin B, Meissner T, Pollok B. The relation between awareness of personal resources and metabolic control in children and adolescents with type 1 diabetes. Pediatr Diabetes. 2014. doi:10.1111/pedi.12177. 10.1111/pedi.12177 25040238

[60] King PS, Berg CA, Butner J, Butler JM, Wiebe DJ (2013). Longitudinal Trajectories of Parental Involvement in Type 1 Diabetes and Adolescents’ Adherence.

[61] Serbin LA, Hubert M, Hastings PD, Stack DM, Schwartzman AE (2014). The influence of parenting on early childhood health and health care utilization. J Pediatr Psychol.

[62] Van Allen J, Kuhl ES, Filigno SS, Clifford LM, Connor JM, Stark LJ (2014). Changes in parent motivation predicts changes in body mass index z-score (zBMI) and dietary intake among preschoolers enrolled in a family-based obesity intervention. J Pediatr Psychol.

[63] Moreira H, Frontini R, Bullinger M, Canavarro MC (2013). Caring for a child with type 1 diabetes: links between family cohesion, perceived impact, and parental adjustment. J Fam Psychol.

[64] Horsch A, McManus F, Kennedy P, Edge J (2007). Anxiety, depressive, and posttraumatic stress symptoms in mothers of children with type 1 diabetes. J Trauma Stress.

[65] Ziegler R, Wiedebusch S, Muthny FA (2007). ‘Chronic sorrow’ in parents of children with type-1 diabetes. Pediatr Diabetes.

[66] Faulkner MS, Clark FS (1998). Quality of life for parents of children and adolescents with type 1 diabetes. Diabetes Educ.

[67] Mackey ER, Struemph K, Powell PW, Chen R, Streisand R, Holmes CS (2014). Maternal Depressive Symptoms and Disease Care Status in Youth with Type 1 Diabetes.

[68] Butwicka A, Zalepa A, Fendler W, Szadkowska A, Mlynarski W (2013). Maternal depressive symptoms predict acute hospitalization among children with type 1 diabetes. Pediatr Diabetes.

[69] Wiebe DJ, Chow CM, Palmer DL, Butner J, Butler JM, Osborn P (2014). Developmental processes associated with longitudinal declines in parental responsibility and adherence to type 1 diabetes management across adolescence. J Pediatr Psychol.

[70] Preacher KJ, Hayes AF (2008). Asymptotic and resampling strategies for assessing and comparing indirect effects in multiple mediator models. Behav Res Methods.

[71] Pope C, Ziebland S, Mays N (2000). Analysing qualitative data. BMJ.

[72] Gale NK, Heath G, Cameron E, Rashid S, Redwood S (2013). Using the framework method for the analysis of qualitative data in multi-disciplinary health research. BMC Med Res Methodol.

[73] Northam EA, Matthews LK, Anderson PJ, Cameron FJ, Werther GA (2005). Psychiatric morbidity and health outcome in type 1 diabetes-perspectives from a prospective longitudinal study. Diabet Med.

[74] Holmbeck GN, Shapera W, Kendall PC, Butcher JN, Holmbeck GN (1999). Research Methods with Adolescents. Handbook of Research Methods in Clinical Psychology.

[75] Goodman R (1997). The strengths and difficulties questionnaire: a research note. J Child Psychol Psychiatry.

[76] Ravens-Sieberer U, Bullinger M. The revised KINDL-R: final results on reliability, validity and responsiveness of a modular HRQOL instrument for children and adolescents. Qual Life Res. 2001;199.

[77] Ravens-Sieberer U, Gosch A, Rajmil L, Erhart M, Bruil J, Power M (2008). The KIDSCREEN-52 quality of life measure for children and adolescents: psychometric results from a cross-cultural survey in 13 European Countries. Value Health.

[78] Ravens-Sieberer U, Herdman M, Devine J, Otto C, Bullinger M, Rose M, et al. The European KIDSCREEN approach to measure quality of life and well-being in children: development, current application, and future advances. Qual Life Res. 2013;1–13. 10.1007/s11136-013-0428-3 PMC3953538 23686556

[79] Anderson BJ, Auslander WF, Jung KC, Miller JP, Santiago JV (1990). Assessing family sharing of diabetes responsibilities. J Pediatr Psychol.

[80] de Wit M, Winterdijk P, Aanstoot HJ, Anderson BJ, Danne T, Deeb L (2012). Assessing diabetes-related quality of life of youth with type 1 diabetes in routine clinical care: the MIND Youth Questionnaire (MY-Q). Pediatr Diabetes.

[81] Van Der Ven N, Weinger K, Yi J, Pouwer F, Ader H, Van Der Ploeg HM (2003). The confidence in diabetes self-care scale: psychometric properties of a new measure of diabetes-specific self-efficacy in Dutch and US patients with type 1 diabetes. Diabetes Care.

[82] Weissberg-Benchell J, Glasgow AM, Tynan WD, Wirtz P, Turek J, Ward J (1995). Adolescent diabetes management and mismanagement. Diabetes Care.

[83] Wechsler D (1991). WISC-III: Wechsler Intelligence Scale for Children.

[84] Wechsler D (2002). WISC III Handleiding.

[85] Fan J, McCandliss BD, Fossella J, Flombaum JI, Posner MI (2005). The activation of attentional networks. NeuroImage.

[86] de Kieviet JF, van Elburg RM, Lafeber HN, Oosterlaan J (2012). Attention problems of very preterm children compared with age-matched term controls at school-age. J Pediatr.

[87] Eriksen CW (1995). The flankers task and response competition: a useful tool for investigating a variety of cognitive problems. Vis Cogn.

[88] Aarnoudse-Moens CS, Duivenvoorden HJ, Weisglas-Kuperus NYNK, Van Goudoever JB, Oosterlaan J (2012). The profile of executive function in very preterm children at 4 to 12 years. Dev Med Child Neurol.

[89] Westerberg H, Hirvikoski T, Forssberg H, Klingberg T (2004). Visuo-spatial working memory span: a sensitive measure of cognitive deficits in children with ADHD. Child Neuropsychology.

[90] Nutley SB, Soderqvist S, Bryde S, Humphreys K, Klingberg T (2010). Measuring working memory capacity with greater precision in the lower capacity ranges. Dev Neuropsychol.

[91] Gioia GA, Isquith PK, Retzlaff PD, Espy KA (2002). Confirmatory factor analysis of the behavior rating inventory of executive function (BRIEF) in a clinical sample. Child Neuropsychology.

[92] Gioia GA, Isquith PK, Guy SC, Kenworthy L (2000). Behavior rating inventory of executive function. Child Neuropsychology.

[93] Markowitz JT, Volkening LK, Antisdel-Lomaglio J, Anderson BJ, Laffel LM (2012). Re-examining a measure of diabetes-related burden in parents of young people with type 1 diabetes: the Problem Areas in Diabetes Survey - Parent Revised version (PAID-PR). Diabet Med.

[94] Antisdel JE (2001). Diabetes-specific distress among parents of youth with type 1 diabetes (Abstract). Diss Abstr Int.

[95] de Wit M, Pouwer F, Gemke RJBJ, Delemarre-van ae Waal H, Snoek FJ (2007). Validation of the WHO-5 well-being index in adolescents with type 1 diabetes. Diabetes Care.

[96] Lowe B, Spitzer RL, Grafe K, Kroenke K, Quenter A, Zipfel S (2004). Comparative validity of three screening questionnaires for DSM-IV depressive disorders and physicians’ diagnoses. J Affect Disord.

[97] Bech P, Gudex C, Johansen KS (1996). The WHO (Ten) well-being index: validation in diabetes. Psychother Psychosom.

[98] Schafer LC, McCaul KD, Glasgow RE (1986). Supportive and nonsupportive family behaviors: relationships to adherence and metabolic control in persons with type i diabetes. Diabetes Care.

