# Supplementary material for: Diabetes IN develOpment (DINO): the bio-psychosocial, family functioning and parental well-being of youth with type 1 diabetes: a longitudinal cohort study design
Source: BMC Pediatr. 2015 Jul 15;15:82. doi: 10.1186/s12887-015-0400-1 (PMC4502615; doi:10.1186/s12887-015-0400-1)
Supplement: Additional file 1: — Study measures online text supplement. [file 12887_2015_400_MOESM1_ESM.docx]

*Study measures online text supplement*

*Biological development*

To assess biological development, in addition to information provided by the pediatric diabetes team self report measures are used. The biological development is assessed both with respect to socio-demographic and clinical data.

Socio-demographic data cover: date of birth and gender are documented at baseline by the diabetes teams. Ethnicity, family structure, education (school level) and family-related life events are evaluated every year by the parents. The latter refers to either positive or negative life events that can impact the adolescent and his/her family (e.g. birth, marriage, divorce, moving house, death of a relative etc.). By classifying the highest level of education in a household, social economic status is determined.

Clinical data (annually documented by the diabetes care team) include: history of medical and psychological (contact with mental health services) co-morbidity; treatment regimen (number of injections/pump, insulin units); care consumption (referrals, nr. of contacts with pediatrician, nurse, psychologist etc.); Tanner stage (pubertal stages will be determined by visual inspection, using the criteria and definitions described by Tanner [1] and testes volume of boys will be determined by palpation); blood pressure; weight; height; glycemic control (determined by means of glycosylated hemoglobin (HbA_1c_)); number of diabetes-related hospitalizations; diabetes ketoacidosis (DKA); indicators for complications (micro-albumin excretion rate, lipid profile and retina screening). Although we do not measure the quality of care, we gain insight in the care consumption as an indicator for what is optimal care. Since all assessments are scheduled in routine care, no extra assessments need to be scheduled. The presence or absence of severe hypoglycemic episodes (defined as needing more assistance from others than usual) are evaluated every year by the parents.

*Study measures – Psychosocial development*Psychosocial functioning of youth 11 years and older is assessed annually by using 7 questionnaires combined in one online survey.

*Emotional and behavioral functioning*: The Strengths and Difficulties Questionnaire (SDQ) [2,3] is used to measure emotional and behavioral functioning and contains 25 items, rated on a 3-point Likert scale. The SDQ comprises five scales: Emotional symptoms, Conduct problems, Hyperactivity/inattention, Peer relationship problems and Pro-social behavior. Cronbach’s α for parents is 0.81, for self-report 0.70 [2,3]. Both adolescents and parents complete the SDQ.

*Self esteem*: The KINDL-R self esteem subscale (4 items on a 5 point Likert scale) assesses overall feelings of self-worth and self-acceptance [4]. Cronbach’s α for the total KINDL-R is 0.82 [5].

*Autonomy*: The Autonomy subscale of the KIDSCREEN asks about generic autonomy with 5 items on a 5 point Likert scale. Cronbach’s α for this subscale is 0.84 [6,7].

*Deviation responsibility diabetes tasks*: Diabetes Family Responsibility Questionnaire (DFRQ) (17 items) is used to examine the transfer of responsibility for diabetes management tasks from parents to youth [8]. On each item is asked who initiates the responsibility: the parent, both parent and child equally or the child. Both adolescents and parents complete the DFRQ. Internal consistency has been reported and considered acceptable [8]

*Quality of Life*: Diabetes related QoL and Diabetes Self management are assessed by the MIND Youth Questionnaire (MY-Q) [9], in which the following domains are covered: social impact (friends, school, free time), parents, responsibility, body image and eating behavior, control perceptions, worries, treatment satisfaction and emotional well-being. Cronbach’s α of the MY-Q is 0.8 [9]

*Self-efficacy*: an adapted version of the Confidence in Diabetes Self Care Scale (CIDS) suitable for adolescents is used to measure diabetes specific self-efficacy [10]. The original adult version of the CIDS consists of 20 items on a 5 point Likert scale and Cronbach’s α=0.86 [10]. This adapted youth version consists of 12 items: 10 of the original, 2 items combined to 1 (original question 2 and 20) and 1 additional item regarding alternations in blood glucose.

*Management in diabetes self-care*: This is assessed by using a renewed version of the diabetes mismanagement scale [11]. The original version consists of 10 items, 3 items were used and 1 was adjusted, recall is changed from 10 days into 7 days. Answers are given on a 5 point Likert scale. Cronbach’s α of the original version is 0.74 and 0.60, respectively [11].

*Adherence*: Diabetes teams are annually asked about their opinion with regard to adolescent’s overall diabetes adherence and diabetes instructions.

Psychological functioning of children 8-10 is assessed by a selection of these questionnaires (KINDL, adapted (shorter and easier to read) version of the MY-Q and KIDSCREEN), as not all are validated for younger children.

*Disturbed eating behavior*: DEB is assessed in adolescents 11 years and older in a step-wise manner in order to minimize the burden in adolescents with no DEB and younger participants. Diabetes specific measures are used as it has been shown that generic measures tend to inflate estimates of eating problems in a population with T1D [12,13].

Step 1: The MY-Q subscale Body image & eating behavior serves as a start asking general questions about trying to control weight, body image, eating binges and skipping insulin. In addition, two questions about dieting status and dieting frequency are asked [14].

Step 2: If any of these items are endorsed, the next step is to assess diabetes specific DEB and weight control behavior by using the Diabetes Eating Problems Scale-Revised (DEPS-R) [15,16] and questions of the AHEAD study [14]. DEPS-R is specifically designed to measure with 16 items on a 6 point Likert scale eating problems in the context of diabetes, including insulin restriction. Cronbach’s α = 0.86 [15,16]. Adolescents can endorse on AHEAD weight control items whether certain behavior (e.g. ate less high-fat foods) occurred in the past 3 months [14].

Step 3: Additionally, adolescents enrolled in step 2 are invited to participate in interviews. Our aim is to gain better insight in initiation of DEB, pro’s and con’s, insulin restriction (maladaptive, treatment (un-)related), course of DEB and receptiveness to help. In order to assess whether parents think their child shows disturbed eating behavior, the parental online survey includes two adjusted questions from the MY-Q regarding weight and looks (Do you have the impression your child is happy with the way he/she looks? Do you have the impression your child’s tries to control her weight by eating less or omitting insulin?).

*Study measures – Cognitive development*Cognitive development is assessed annually using a short form of the Wechsler Intelligence Scale for Children-III (WISC-III) [17], three computerized tasks, and an executive functioning questionnaire completed by parents. Ideally assessment takes place prior or following the adolescent’s regular assessment with the diabetes team in the diabetes clinic. Prior, during and after cognitive assessment, blood glucose values are measured. If the blood glucose level is found below or above the adolescent’s personal average (ideally between 5-10) and participant’s concentration is therefore impaired, examination is postponed until blood glucose is considered normal. In statistical analyses we will correct for extreme low or high blood glucose values. Tests were administered in fixed order by trained examiners using standardized instructions.

Full scale IQ will be estimated using a well-established short version of the Wechsler Intelligence Scale for Children (WISC-III), using five subtests: Information; Picture arrangement; Arithmetic; Block design; Digit Span [17-19].

With respect to the computerized tasks, an adapted version of the Posner’s Attention Network Task (ANT) [20,21] suitable for children is used to assess orienting, alerting and executive attention, as well as speed and consistency in speed of information processing. The Eriksen Flanker Task [22,23] is used to assess interference control. An adapted version of the Klingberg task [21,24,25] is used to measure visuo-spatial working memory.

Executive functioning is additionally measured using the Behavior Rating Inventory of Executive Functioning questionnaire (BRIEF) [26,27], an 86 item 3-point Likert scale parent report questionnaire. The BRIEF allow calculation of two indices: the Behavioral Regulation index (subdomains Inhibition, Shift and Emotional Control) and Metacognition index (subdomains Initiate, Working Memory, Plan/Organize, Organization of materials, and Monitor) [27].

Performance on the WISC-III and BRIEF will be compared to normative data. Computerized measures will be administered to a gender and age matched sample of 100 healthy controls as there is currently no Dutch normative sample is available for these tasks. Healthy controls are derived from primary and secondary schools in the Netherlands and will complete identical cognitive tests as the diabetes group (including the subtasks of the WISC III) and parents will complete the BRIEF. Controls will be measured cross-sectional, at baseline.

*Study measures – Parental assessment*

To measure family functioning and parental well-being, parents annually complete an online survey in which they are asked about their child’s development and their own well-being and parenting style. The latter is assessed by using the following measures.

Parental distress: Problem Areas In Diabetes-Parents Revised (PAID-PR) assesses diabetes related distress in parents of children with diabetes in 20 items, scored on a 5 point Likert scale. Cronbach’s α=0.87 [28,29].

Parental well-being: WHO-Five Well-being Index (WHO-5) captures emotional well-being with five positively worded items, scored on a 6 point Likert scale. Cronbach’s α=0.91 [30-32].

Diabetes specific parenting style: The Diabetes Family Behavior Checklist (DFBC) [33] assesses the amount of supportive and non-supportive diabetes specific parenting behavior. DFBC contains 16 items on a 5 point Likert scale, of which nine regarding positive parenting behaviors and seven negative. Cronbach’s α for the positive items is 0.73, for the negative 0.43 [33]. Parents decide themselves whether the mother or the father complete the online survey. We expect that the majority will be mothers.

Reference List

1. Tanner JM: **Normal growth and techniques of growth assessment.** *Clin Endocrinol Metab* 1986, **15:** 411-451.

2. van Widenfelt BM, Goedhart AW, Treffers PD, Goodman R: **Dutch version of the Strengths and Difficulties Questionnaire (SDQ).** *Eur Child Adolesc Psychiatry* 2003, **12:** 281-289.

3. Goodman R: **The Strengths and Difficulties Questionnaire: a research note.** *J Child Psychol Psychiatry* 1997, **38:** 581-586.

4. Ravens-Sieberer U, Bullinger M: **The revised KINDL-R: Final results on reliability, validity and responsiveness of a modular HRQOL instrument for children and adolescents.** *Quality of Life Research* 2001, 199.

5. Ravens-Sieberer U, Bettge S, Erhart M: **Lebensqualitat von Kindern und Jugendlichen-Ergebnisse aus der Pilotphase des Kinder-und Jugendgesundheitssurveys.** *Bundesgesundheitsblatt-Gesundheitsforschung-Gesundheitsschutz* 2003, **46:** 340-345.

6. Ravens-Sieberer U, Gosch A, Rajmil L, Erhart M, Bruil J, Power M *et al*.: **The KIDSCREEN-52 Quality of Life Measure for Children and Adolescents: Psychometric Results from a Cross−Cultural Survey in 13 European Countries.** *Value in Health* 2008, **11:** 645-658.

7. Ravens-Sieberer U, Herdman M, Devine J, Otto C, Bullinger M, Rose M *et al*.: **The European KIDSCREEN approach to measure quality of life and well-being in children: development, current application, and future advances.** *Quality of Life Research* 2013, 1-13.

8. Anderson BJ, Auslander WF, Jung KC, Miller JP, Santiago JV: **Assessing family sharing of diabetes responsibilities.** *J Pediatr Psychol* 1990, **15:** 477-492.

9. de Wit M, Winterdijk P, Aanstoot HJ, Anderson BJ, Danne T, Deeb L *et al*.: **Assessing diabetes-related quality of life of youth with type 1 diabetes in routine clinical care: the MIND Youth Questionnaire (MY-Q).** *Pediatric Diabetes* 2012, **13:** 1-9.

10. Van Der Ven N, Weinger K, Yi J, Pouwer F, Ader H, Van Der Ploeg HM *et al*.: **The confidence in diabetes self-care scale: psychometric properties of a new measure of diabetes-specific self-efficacy in Dutch and US patients with type 1 diabetes.** *Diabetes Care* 2003, **26:** 713-718.

11. Weissberg-Benchell J, Glasgow AM, Tynan WD, Wirtz P, Turek J, Ward J: **Adolescent diabetes management and mismanagement.** *Diabetes Care* 1995, **18:** 77-82.

12. Young V, Eiser C, Johnson B, Brierley S, Epton T, Elliott J *et al*.: **Eating problems in adolescents with Type 1 diabetes: a systematic review with meta−analysis.** *Diabetic medicine* 2013, **30:** 189-198.

13. Young-Hyman DL, Davis CL: **Disordered Eating Behavior in Individuals With Diabetes: Importance of context, evaluation, and classification.** *Diabetes Care* 2010, **33:** 683-689.

14. Neumark-Sztainer D, Patterson J, Mellin A, Ackard DM, Utter J, Story M *et al*.: **Weight Control Practices and Disordered Eating Behaviors Among Adolescent Females and Males With Type 1 Diabetes Associations with sociodemographics, weight concerns, familial factors, and metabolic outcomes.** *Diabetes Care* 2002, **25:** 1289-1296.

15. Markowitz JT, Butler DA, Volkening LK, Antisdel JE, Anderson BJ, Laffel LM: **Brief screening tool for disordered eating in diabetes internal consistency and external validity in a contemporary sample of pediatric patients with type 1 diabetes.** *Diabetes Care* 2010, **33:** 495-500.

16. Wisting L, Froisland DH, Skrivarhaug T, Dahl-Jorgensen K, Ro O: **Disturbed Eating Behavior and Omission of Insulin in Adolescents Receiving Intensified Insulin Treatment A nationwide population-based study.** *Diabetes Care* 2013, **36:** 3382-3387.

17. Wechsler D: *WISC-III: Wechsler intelligence scale for children*. Psychological Corporation San Antonio, TX; 1991.

18. Sattler JM: **Assessment of children's intelligence.** *Philadelphia, PA: WB Saunders* 1974.

19. Wechsler D: *WISC III Handleiding*. London, United Kingdom: The Psychological Corporation; 2002.

20. Fan J, McCandliss BD, Fossella J, Flombaum JI, Posner MI: **The activation of attentional networks.** *NeuroImage* 2005, **26:** 471-479.

21. de Kieviet JF, van Elburg RM, Lafeber HN, Oosterlaan J: **Attention problems of very preterm children compared with age-matched term controls at school-age.** *The Journal of pediatrics* 2012, **161:** 824-829.

22. Eriksen CW: **The flankers task and response competition: A useful tool for investigating a variety of cognitive problems.** *Visual Cognition* 1995, **2:** 101-118.

23. Aarnoudse-Moens CS, Duivenvoorden HJ, Weisglas-Kuperus NYNK, Van Goudoever JB, Oosterlaan J: **The profile of executive function in very preterm children at 4 to 12 years.** *Developmental Medicine & Child Neurology* 2012, **54:** 247-253.

24. Westerberg H, Hirvikoski T, Forssberg H, Klingberg T: **Visuo-Spatial Working Memory Span: A Sensitive Measure of Cognitive Deficits in Children With ADHD.** *Child Neuropsychology* 2004, **10:** 155-161.

25. Nutley SB, Soderqvist S, Bryde S, Humphreys K, Klingberg T: **Measuring working memory capacity with greater precision in the lower capacity ranges.** *Developmental neuropsychology* 2010, **35:** 81-95.

26. Gioia GA, Isquith PK, Retzlaff PD, Espy KA: **Confirmatory Factor Analysis of the Behavior Rating Inventory of Executive Function (BRIEF) in a Clinical Sample.** *Child Neuropsychology* 2002, **8:** 249-257.

27. Gioia GA, Isquith PK, Guy SC, Kenworthy L: **Behavior Rating Inventory of Executive Function.** *Child Neuropsychology* 2000, **6:** 235-238.

28. Antisdel JE: **Diabetes-specific distress among parents of youth with type 1 diabetes (Abstract).** *Dissertation Abstracts International* 2001, **61:6123**.

29. Markowitz JT, Volkening LK, Antisdel-Lomaglio J, Anderson BJ, Laffel LM: **Re-examining a measure of diabetes-related burden in parents of young people with Type 1 diabetes: the Problem Areas in Diabetes Survey - Parent Revised version (PAID-PR).** *Diabet Med* 2012, **29:** 526-530.

30. de Wit M, Pouwer F, Gemke RJBJ, Delemarre-van de Waal H, Snoek FJ: **Validation of the WHO-5 Well-Being Index in adolescents with type 1 diabetes.** *Diabetes Care* 2007, **30:** 2003-2006.

31. Lowe B, Spitzer RL, Grafe K, Kroenke K, Quenter A, Zipfel S *et al*.: **Comparative validity of three screening questionnaires for DSM-IV depressive disorders and physicians' diagnoses.** *Journal of Affective Disorders* 2004, **78:** 131-140.

32. Bech P, Gudex C, Johansen KS: **The WHO (Ten) Well-Being Index: validation in diabetes.** *Psychother Psychosom* 1996, **65:** 183-190.

33. Schafer LC, McCaul KD, Glasgow RE: **Supportive and Nonsupportive Family Behaviors: Relationships to Adherence and Metabolic Control in Persons with Type I Diabetes.** *Diabetes Care* 1986, **9:** 179-185.
